# Supplementary material for: Nucleotide-Binding Oligomerization Domain 1 (NOD1) Agonists Prevent SARS-CoV-2 Infection in Human Lung Epithelial Cells through Harnessing the Innate Immune Response
Source: Int J Mol Sci. 2024 May 13;25(10):5318. doi: 10.3390/ijms25105318 (PMC11121681; doi:10.3390/ijms25105318)
Supplement: Supplementary file 1 [file ijms-25-05318-s001.zip › ijms-2974164-supplementary.pdf]

Supplementary Materials

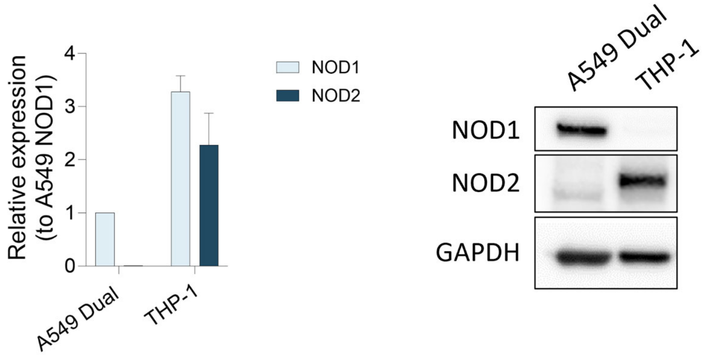

Supplementary Figure S1. NLRs expression in lung epithelial A549-Dual and myeloid THP-1 cells. (**left**) Gene expression (mRNA) of NOD1 and NOD2 receptors in A549-Dual and THP-1 cells, as measured by RT-qPCR. Values were relativized to the expression of NOD1 in A549-Dual cells. (**right**) Expression of NOD1 and NOD2 receptors at protein level in lung epithelial A549-Dual and myeloid THP-1 cells. Representative western blot (out of three) showing the expression of NOD1 and NOD2 is shown.

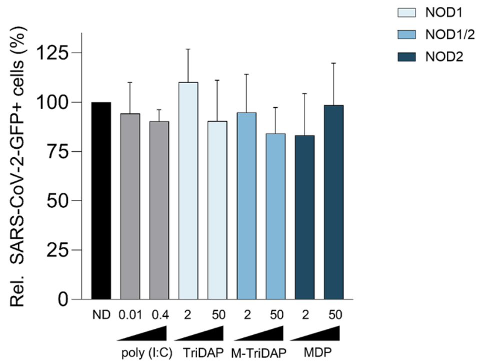

Supplementary Figure S2. Assessment of NOD antiviral activity in Vero E6 cells. Pretreatment of IFN-deficient Vero E6 cells for 3 h with increasing concentrations of NOD1 and dual NOD1/2 agonists did not inhibit SARS-CoV-2 replication. Viral replication was measured as the percentage of SARS-CoV-2-GFP+ cells determined by flow cytometry after 24 h of infection. Values were relativized to the untreated condition (INF, black filled bar). TLR3 agonist Poly (I:C) (light grey bars) was used as control. Mean ± SD of three independent experiments is shown.

Supplementary Table S1. Summary of Immunomodulators used in the study

Compound name	Target	Activity
G140	cGAS-STING	Inhibitor
H-151	cGAS-STING	Antagonist
MRT67307	IKKε /TBK1	Inhibitor
Pepinh-Control	Peptides	Control
Pepinh-MYD	MyD88	Inhibitor
Pepinh-TRIF	TRIF	Inhibitor
3p-hpRNA	RIG-I	Agonist
BX795	IKKε /TBK1	Inhibitor
BAY 11-7082	NF-κB/NLRP3	Inhibitor
G3-YSD	cGAS-STING	Agonist
G3-YSDcontrol	cGAS-STING	Control
Imiquimod	TLR7	Agonist
CL075	TLR7/8	Agonist
ODN2006	TLR9	Agonist
ODN2006control	ODN2006 [TLR9]	Control
ODN2216	TLR9	Agonist
ODN2216control	ODN2216 [TLR9]	Control
ODN2395	TLR9	Agonist
ODN2395control	ODN2395 [TLR9]	Control
R848	TLR7/8	Agonist
ssRNA40	TLR8	Agonist
ssRNA41	TLR8	Agonist
C12-iE-DAP	NOD1	Agonist
iE-DAP	NOD1	Agonist
L18-MDP	NOD2	Agonist
MDP	NOD2	Agonist
M-TriDAP	NOD1/2	Agonist
M-TriLYS	NOD2	Agonist
Murabutide	NOD2	Agonist
PGN-ECndi	NOD1/2	Agonist
PGN-Sandi	NOD1/2	Agonist
Tri-DAP	NOD1	Agonist
CU-CPT9a	TLR8	Inhibitor
HSV60	DDX41/IFI16 [STING]	Agonist
HSV60control	DDX41/IFI16 [STING]	Control
Indirubin	AhR	Agonist
L-Kynurenine	AhR	Agonist
ODN2088	TLR9	Antagonist
PolydA:dT	cGAS-STING/RIG-I	Agonist
PolydG:dC	cGAS-STING/	Agonist
PolyA:U	TLR3	Agonist
PolyI:C (HMV)	TLR3	Agonist
PolyI:C(LMW)	TLR3	Agonist
VACV70	IFI16 [STING]	Agonist
VACV70control	IFI16 [STING]	Control

* AhR; Aryl hydrocarbon receptor.
